# Small subcortical ischemic infarction and other DWI lesions establish predictive model for MES

**DOI:** 10.3389/fneur.2024.1519894

**Published:** 2025-01-13

**Authors:** Liming Zhao, Yicheng Xu, Hongqin Zhao, Senlin Wang, Jiatang Zhang, Chenglin Tian, Aijuan Zhang, Zengchao Zhang, Tailing Ji, Zhengang Wang

**Affiliations:** ^1^Department of Neurology, Affiliated Hospital of Shandong Second Medical University, Weifang, China; ^2^Department of Neurology, Aerospace Center Hospital, Beijing, China; ^3^Department of Neurology, Affiliated Hospital of Qingdao University, Qingdao, China; ^4^Department of Neurology, The Second People's Hospital of Weifang, Weifang, China; ^5^Department of Neurology, Chinese People's Liberation Army General Hospital, Beijing, China; ^6^Department of Neurology, Weifang People's Hospital, Weifang, China; ^7^Department of Neurosurgery, Affiliated Hospital of Shandong Second Medical University, Weifang, China

**Keywords:** microembolic signals (MES), lesion pattern, subcortical infarction, nomogram, border zone (BZ)

## Abstract

**Objective:**

The relationship between small subcortical ischemic infarction remains poorly characterized. Therefore, the present study aimed to investigate the association between artery-to-artery embolization and small subcortical infarctions.

**Methods:**

This retrospective observational cross-sectional study enrolling 230 patients with acute middle cerebral artery (MCA) stroke classified into the microembolic signals-positive (MES+) and MES-negative (MES−) groups. The diffusion weighted imaging (DWI) infarction patterns in the MCA were divided into the territorial, border zone (BZ), cortical, and subcortical infarcts. We set the standard of small subcortical infarction (SCI) into two levels: < 10 mm diameter and <5 mm diameter. Relevant DWI parameters were used to build a nomogram for MES+, using free statistics.

**Results:**

MES occurred in 38 of the 230 cases, yielding a positivity rate of 16.5%. BZ, SCI <10 mm, cortical ischemia (CI), stenosis, white blood cell count, and gender were compared between the MES+ and MES− groups. Multivariate analysis revealed that BZ, SCI < 10 mm, and CI were independently associated with MES. Based on DWI parameters, a nomogram model was built for MES+. The area under the curve of the model was 0.826 (95%CI 0.764 to 0.889). In internal cross-validation, the slope of the calibration curve was 1.000, indicating that the model accurately predicted unsuccessful treatment outcomes.

**Conclusion:**

Small subcortical infarctions are associated with MES. In the present study, we built a predictive nomogram model for MES+ based on small subcortical infarctions and other DWI parameters. This model demonstrated good performance in clinical practice.

## 1 Introduction

Ischemic stroke is the leading cause of death and disability in adults worldwide. The incidence of major atherosclerosis (LAA) in East Asia is higher than that of other ischemic stroke etiologies (1). Artery-to-artery embolization is one of three primary mechanisms underlying LAA (2, 3). Microembolics are associated with plaque destabilization (4). Microembolic signals (MES) detected by transcranial Doppler ultrasound (TCD) represent the most direct evidence of an artery-to-artery embolization mechanism in LAA stroke, and are related with recurrent stroke (5–7).

Diffusion-weighted imaging (DWI) lesions patterning has been shown to be related with the mechanisms of stroke and recurrence (5, 8–12). Previous studies have shown that border zones and cortical infarctions often coexist, and are associated with MES (2, 13, 14). However, relatively little is known about the relationship between small subcortical ischemia (SCI) and MES.

SCI and lacunar infarction, which are associated with complex underlying mechanisms, account for nearly one-third of all cases of ischemic strokes (15–17). Due to this heterogeneity in SCI mechanisms, controversies exist between the TOAST and CISS stroke subclassification guidelines (18, 19). One prior study showed that a considerable proportion of small SCI may be associated with LAA (20). However, to the best of our knowledge, there is currently no evidence to prove the relationship between small SCI and artery-to-artery (A-A) embolization (21).

Therefore, the present study aimed to explore the relationship between small SCI and MES. Furthermore, we combined other lesion patterns to build a DWI radiomic model to assess A-A embolisms.

## 2 Methods

### 2.1 Patients and study design

This was a retrospective observational cross-sectional study from two medical centers that together provide health care for a total population of more than 20 million individuals in the Shandong province of China. The first institute was the Department of Neurology at Weifang Brain Hospital, which was enrolled in the present study from January 2015 to October 2019, and the second was the Department of Neurology at the Affiliated Hospital of Qingdao University, which was enrolled in the present study from January 2014 to December 2016. Our study was approved by Affiliated Hospital of Shandong Second Medical University Ethics Committee. The approval no. of the Ethics Committee was wyfy-2024-ky-495.

Herein, we investigated the relationship between DWI infarction patterns and MES lasting 60 min during TCD monitoring within 72 h after the onset of acute stroke Patients with consecutive acute ischemic stroke within the middle cerebral artery (MCA) territory. Stroke was diagnosed based on the imaging characteristics obtained via magnetic resonance imaging (MRI) and neurological deficits lasting for longer than 24 h. The requirement for informed consent was waived. The patients’ general data, relevant medical histories, treatments, and laboratory examinations were evaluated and recorded by a neurologist.

The exclusion criteria for candidate patients were as follows: (1) younger than 40 years old; (2) carotid artery occlusion or middle cerebral artery occlusion; (3) absence of a temporal acoustic window for TCD monitoring; (4) bilateral anterior infarctions and/or anterior-and posterior-circulation infarctions; (5) cardioembolic stroke, or strokes with etiologies differing from circulation ischemic stroke; (6) severe nephritis or liver disease, or definitive/suspected cancer; (7) no enduring MES for 60 min during TCD monitoring; or (8) a history of carotid endarterectomy or a carotid artery stent.

### 2.2 Assessment of MES via TCD monitoring

MES was detected via TCD monitoring (Delica EMS-9A), the specific details of which are described in our previous study (22).

### 2.3 DWI infarction pattern

The DWI infarction pattern in the MCA was divided into the territorial, internal border zone (BZ), cortical, and subcortical infarcts, and a correlation was analyzed between the DWI infarction pattern and MES microembolic signals. BZ infarcts were divided into internal and cortical BZ infarcts; of which the latter encompassed infarcts in the Frontal cortex (between the anterior cerebral arteries and middle cerebral arteries) and Occipital cortex (between the middle cerebral arteries and posterior cerebral arteries) (23–25). Based on previous study (26, 27), we set the standards of small subcortical infarction into two levels, as <10 or < 5 mm in diameter (28). The lesion pattern in a single patient may involve the formation of more than one lesion.

### 2.4 Statistical analysis

SPSS (version 22.0; Chicago, IL, United States) and Free Statistics (version 1.7.1) software were used for data analysis. Quantitative data are expressed as the mean ± standard deviation, while qualitative data are expressed as frequencies and percentages. After testing for normality, intergroup comparisons of quantitative data were performed using *t*-tests, and qualitative or categorical data were compared using χ^2^ or Fisher’s exact texts. Statistical significant factors (*p* < 0.05) were analyzed for collinearity before regression analysis. Statistically significant factors in the univariate analyses were included in a stepwise forward logistic regression analysis to identify the independent factors for MES. Odds ratios (ORs) and their 95% CIs were used to evaluate the independent contributions of significant factors. The Hosmer-Lemeshow test was applied to estimate the appropriateness of the model.

A nomogram graph was built using Free Statistics software. Receiver operating characteristic (ROC) curve, concordance index (C-index), and calibration curve analyses were performed to evaluate the discrimination and calibration of the model.

## 3 Results

### 3.1 Baseline demographics

**Figure 1:**
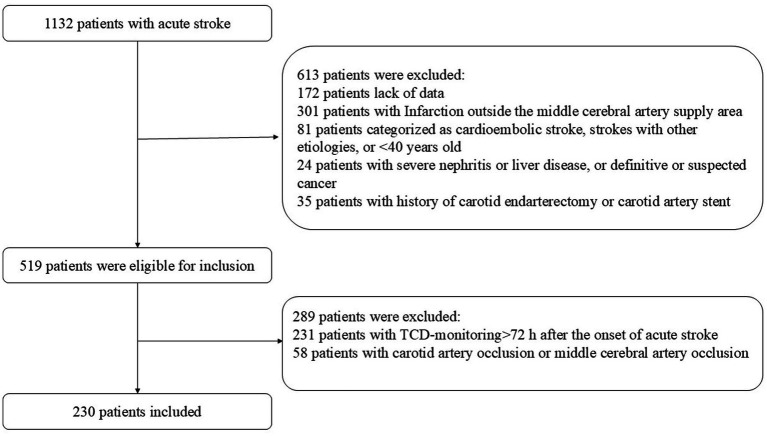
Flowchart of patients included in the present study.

During the study period, 1,132 consecutive patients with acute stroke were deemed eligible. After excluding patients who met the exclusion criteria, 230 patients (149 from the Affiliated Hospital of Qingdao University and 81 from Weifang Brain Hospital)with acute MCA stroke were enrolled in the study (Figure 1). MES occurred in 38 of the 230 cases, with a positivity rate of 16.5%. Male sex, stenosis, and white blood cell counts were significantly higher in the MES+ group than in the MES-group. There were no significant differences in terms of hypertension, diabetes mellitus, ischemic heart disease, stroke history, smoking, drinking, dual antiplatelet, C-reactive platelets, or platelets between MES+ and MES− patients. The general clinical characteristics of the patients are shown in Table 1.

**Table 1:** Baseline demographics.

	Total (230)	MES+ (38)	MES- (192)	T/χ^2^	p
Gender (male)	166 (72.2)	33 (86.8)	133 (69.3)	4.877	0.029
Age (y)	61.93 ± 9.99	64.45 ± 9.03	61.43 ± 10.11	1.707	0.089
HP (n)	160 (69.6)	24(63.2)	136(70.8)	0.883	0.342
DM (n)	63(27.4)	9(23.7)	54(28.1)	0.315	0.692
CAD (n)	64.8(27.8)	8(21.05)	56(29.2)	1.040	0.428
MI (n)	9(3.9)	1(2.6)	8(4.2)	0.000	1.000
Stroke his (n)	54(23.5)	10(26.3)	44(22.9)	0.204	0.667
Smoking (n)	83(36.1)	17(44.7)	66(34.4)	1.447	0.224
Drinking (n)	58(25.2)	12(31.6)	46(24.0)	0.977	0.315
Dual antiplatelet (n)	87(37.8)	10(26.3)	77(40.1)	2.564	0.143
Stenosis (n)	100(43.5)	24(63.2)	76(39.6)	7.174	0.011
CRP (mmol/L)	5.39 ± 9.81	6.50 ± 9.30	5.21 ± 9.92	0.503	0.616
GLU (mmol/L)	6.51 ± 2.65	6.31 ± 2.43	6.54 ± 2.69	0.354	0.742
WBC (10^12^/L)	7.31 ± 1.94	6.66 ± 1.80	7.44 ± 1.96	−2.264	0.025
PLT (10^9^/L)	222.1 ± 60.1	213.8 ± 64.5	225.7 ± 68.9	−0.721	0.472

Stenosis: ipsilateral middle cerebral artery or internal carotid artery stenosis; MES: microembolic signals; CRP:C-reactive protein WBC: White blood cell; PLT: blood platelet; GLU: vein blood glucose.

### 3.2 DWI lesion patterns of MCA

There were 40 territorial infarction (TI), 55 cases BZ cases, 154 SCI cases, and 64 cortical infarction (CI) cases. Among 154 patients with SCI, 78 and 33 had SCIs with a diameter < 10 mm and < 5 mm, respectively.

### 3.3 Multiple collinear analysis of independent variables and multivariable analysis

The variance inflation factors (VIF) of BZ, SCI < 10 mm, CI, stenosis, WBC count, and sex were all less than 2. Multicollinearity was considered nonexistent. BZ, SCI < 10 mm, CI, stenosis, WBC count, and sex were all therefore entered into the logistic regression analysis. Multivariate analysis revealed that the BZ level, CI, and SCI < 10 mm were independent correlation factors for MES. Other factors were not included in this equation (Table 2).

**Table 2:** DWI lesion patterns and MES.

	Total (230)	MES+ (38)	MES- (192)	T/χ2	p
TI (n/%)	40(17.4)	3(7.9)	37 (19.3)	2.857	0.105
BZ (n/%)	55(23.9)	20 (52.6)	35 (18.2)	29.879	0.002
1score		11	29		
2 score		6	5		
3 score		3	1		
SCI (n/%)	154(67.0)	25(65.9)	129(67.2)	0.028	0.853
SCI <10 mm	78(33.9)	23(60.5)	55(28.6)	14.385	0.000
SCI <5 mm	33(60.5)	12(31.5)	21(10.9)	10.998	0.002
CI	64(27.8)	21(60.5)	43(22.4)	17.063	0.000

TI, territorial infarcts; BZ, Border zone infarcts; SCI, subcortical infarcts; CI, cortical infarcts.

As SCI < 5 mm was found to be significantly different between the two groups in the univariate analysis, we replaced SCI < 10 mm with SCI < 5 mm, and found that SCI < 5 mm was also associated with MES, similar to SCI < 10 mm. The results of the binary logistic regression revealed that SCI < 5 mm was not included in the regression equation, *p* > 0.05.

### 3.4 Nomogram construction and validation

**Figure 2:**
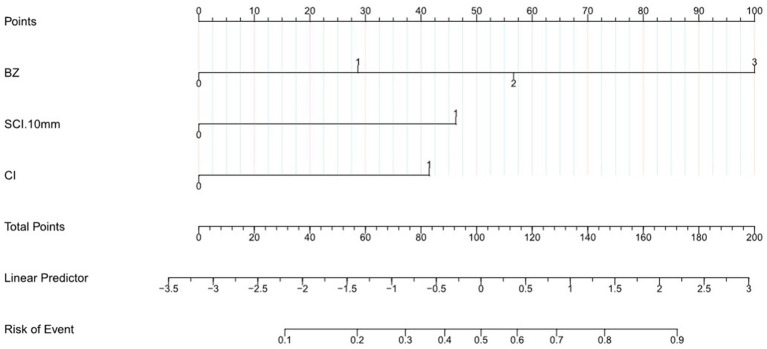
Nomogram to predict the probability of MES in patients with acute stroke of the middle cerebral artery. According to nomogram points for BZ, SCI < 10 mm, CI. The total points were the sum of the three points, and we can evaluate the risk of MES from the line of Risk of Event according to the total points.

**Figure 3:**
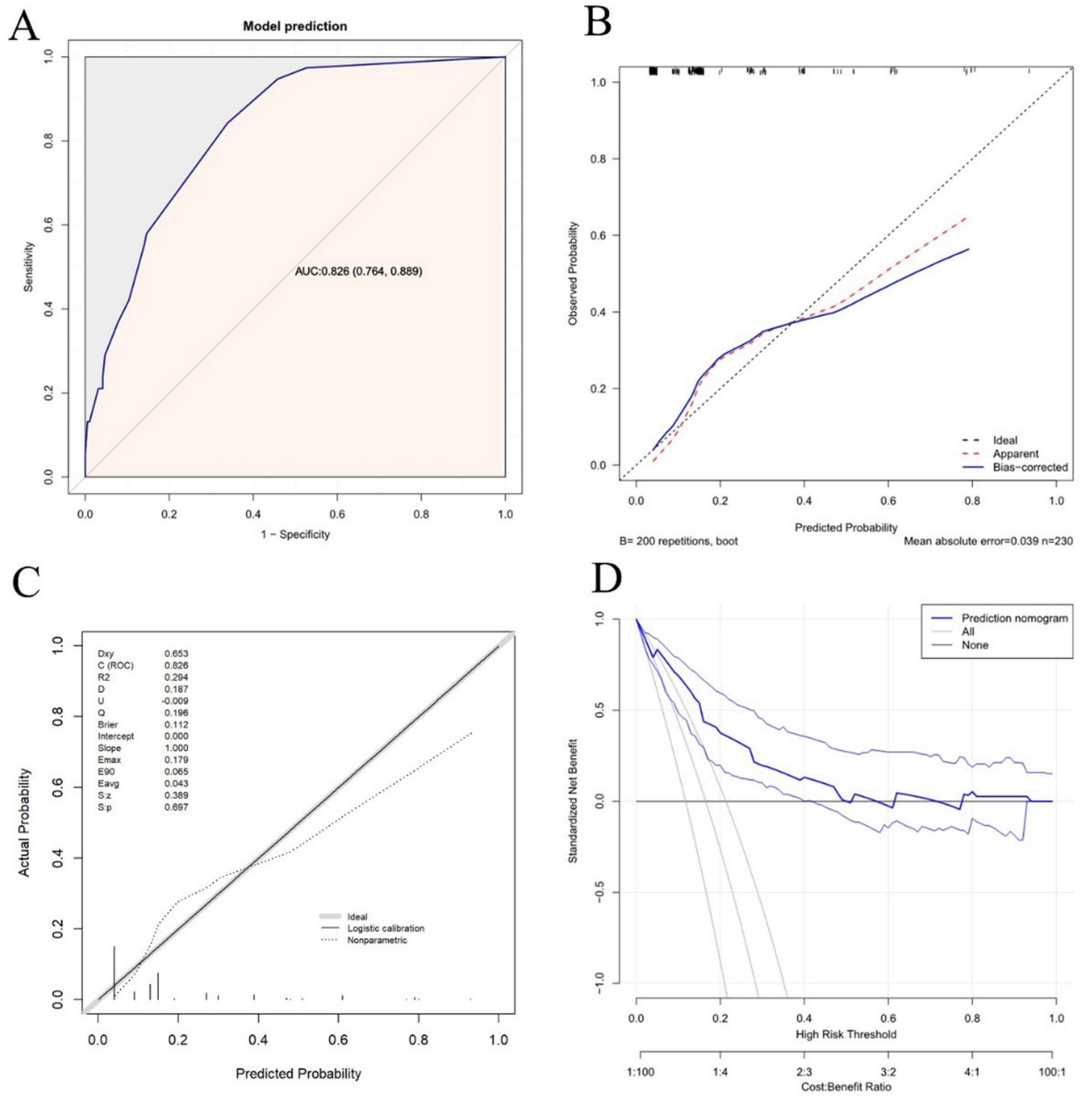
Discrimination and calibration assessment of the model. **(A)** ROC curve and AUC of the nomogram in the training cohort. **(B)** Calibration curve for the nomogram to predict the probability of MES+ with bootstrap sampling validation. The bias-corrected curve is plotted by bootstrapping using 200 resamples. The ideal curve is the 45° dashed line, which indicates perfect prediction. **(C)** Calibration curve for the nomogram to predict the probability of MES+ with leave-one-out cross-validation. **(D)** Decision curve for the predictive nomogram. The net benefits were measured at different threshold probabilities. The blue line represents the predictive nomogram. The middle blue line represents the assumption that all patients are MES+. The middle gray line represents the assumption that all patients are MES–.

Based on the multivariate logistic regression analyses, three independent correlation factors were used to explore the nomogram (Figure 2), which was assessed using area under the curve (AUC), concordance index (C-index), and calibration curve analyses. The AUC for predicting MES+ was 0.826 (95%CI 0.764 to 0.889) (Figure 3A). The accuracy of the model was assessed using a calibration curve, and the slope of the calibration curve was close to the ideal values (Figure 3B). Leave-one-out cross-validation was further performed to evaluate the model (Figure 3C); the corrected C-index was 0.826 and the calibration slope was 1.0. Figure 3D shows the results of the decision curve analysis. This analysis revealed that patients could benefit from the model when the threshold probabilities were approximately 0.1–0.5.

## 4 Discussion

To the best of our knowledge, there have been no prior studies presenting direct and definite research on MES and small SCI. However, the present study, conducted across two medical centers, provided evidence that small SCI (<10 mm) is independently associated with MES, as are CI and BZ infarction, while indiscriminate SCI (irrespective of size or diameter) was not associated with MES. We further developed a simple radiomics prognostic nomogram using DWI parameters, including SCI < 10 mm, CI, and BZ. This nomogram was assessed using the C-index, AUC, and calibration curve, and showed good performance and accuracy in predicting MES+. Based only on DWI parameters, the model would help neurologists conveniently and accurately identify acute MCA stroke with an A-A embolism. For example, a patient’s lesions are distributed in the Frontal BZ, Occipital cortex BZ, subcortex (lesion diameter < 10 mm) and cortex. In the nomograms two BZ score 57 point, SCI < 10 mm score 46 point, CI score 42 point, the total point would be 145 point, which would indicate this patient may have a 80% risk of MES. For patients with higher nomogram scores or high risk of MES, dual antiplatelet therapy may be considered necessary (6) (see Table 3).

**Table 3:** Multiple Collinear Analysis of Independent Variables and Multivariable Analysis.

	OR	95%CI	*p*
BZ	2.548	1.517–4.280	0.000
CI	3.535	1.552–8.054	0.003
SCI < 10 mm	4.235	1.888–9.501	0.000

The pathogenesis of microembolism is usually thought to involve dislodgement of vulnerable plaques by the blood flow, resulting in entry into the distal smaller downstream vessels (29). Whether microembolism can enter a subcortical perforating artery remains unclear. In a study conducted in a monkey model of microembolism, Macdonald concluded that microembolics could enter the subcortical penetrating arteries of monkeys (30). There have been only a few small-sample studies conducted on MES and DWI. One prior study showed that without infarction pattern sub-classification, small infarction (diameter < 10 mm) was associated with MES. The results of our study support this hypothesis. However, it should be noted that this prior study included only 28 patients (27). Another study of SCI and MES in 37 cases found that acute superficial perforator lesions were associated with MES, whereas deep perforator lesions were not (31). However, owing to insufficient sample sizes, these previous studies could not provide evidence to support an independent correlation between small SCI and MES (2, 27, 31), and did not establish a predictive model. A South Korean study on silent stroke and MES after neurointerventional procedures showed that most infarctions occurred in the cortical and border zones. However, the percentage of SCIs was less than one-tenth (28). We believe that silent stroke after the intervention is different from real-world emergency stroke. These silent strokes were tiny infarctions with diameters of less than 5 mm. Nevertheless, these small lesions can still cause neurological deficits. Our research found that DWI infarction of <5 mm represented only a small percentage compared to infarctions <10 mm.

Cortical infarction patterns represent the embolic mechanisms of stroke (5, 28, 31, 32). Hypoperfusion and embolism are the two dominant and coincident pathogeneses of ischemic stroke, commonly associated with severe large artery stenosis (32). The BZ is generally located at the distal intersection of the blood supply to two or more main artery (24). Due to severe large-vessel stenosis or hypovolemia, local cerebral perfusion of the BZ significantly declines, leading to hypoperfusion infarction and impairment of the embolic clearing powder (33). Prior studies have found that the border zone is a region vulnerable to embolism (28). Consistent with previous results, our research showed that cortical and border zone infarction patterns were independent correlation factors for MES.

Large artery stenosis is an important risk factor of MES. Several prior studies have shown that systemic carotid artery stenosis and middle cerebral artery stenosis are related to MES (7, 34). In the current study, large artery stenosis was also associated with MES, but did not show an independent association. We propose that there are several confounding factors, including different collateral circulation blood volumes and plaque vulnerability. Large-vessel atherosclerotic stenosis is the etiopathogenic foundation of both MES and ischemic DWI lesions. DWI lesion patterns may reflect more information and are more closely related to MES than to large-vessel stenosis.

DWI is more sensitive than conventional MRI sequences at identifying new small cerebral ischemic infarctions (35). DWI lesion patterns, including lesion size and distribution, have shown utility in identifying distinct pathophysiological mechanisms and recurrence (8, 10, 12, 36). Differences in the stroke pathophysiology may require different preventive and treatment strategies. Exploring the DWI lesion pattern model of acute MCA stroke is helpful for clinicians in analyzing the mechanism of infarction, evaluating the risk of recurrent stroke, choosing appropriate treatments, and avoiding recurrent stroke.

### 4.1 Strengths and limitations

The present study showed that a small SCI was associated with embolism in two medical center patients. Furthermore, by combining cortical and border-zone infarctions, the study employed small SCI as a factor to build a predictive nomogram model for MES, and these parameters were easily acquired. Third, MES is an important marker of recurrent stroke (7), and this model could provide early clues for stroke recurrence.

Nevertheless, this study has several limitations. First, MES detection was the only method used to identify microemboli, and has some limitations. For example, 1 h of MES-TCD monitoring cannot avoid false-negative bias. Second, the sample size did not satisfy the requirements for external model validation. Finally, this was a retrospective cross-sectional study, and our findings therefore need to be validated in future cohort studies.

## Data Availability

The original contributions presented in the study are included in the article/supplementary material, further inquiries can be directed to the corresponding author.
