# Erratum: Small subcortical ischemic infarction and other DWI lesions establish predictive model for MES

**DOI:** 10.3389/fneur.2025.1571306

**Published:** 2025-02-18

**Authors:** Frontiers Production Office

**Affiliations:** Frontiers Media SA, Lausanne, Switzerland

**Keywords:** microembolic signals (MES), lesion pattern, subcortical infarction, nomogram, border zone (BZ)

Due to a production error, there was a mistake in the legends for **Figures 1, 2, and 3**. Figure 1 used the legend intended for Figure 3, Figure 2 used the legend intended for Figure 1, and Figure 3 used the legend intended for Figure 2. The corrected legends for the three figures are listed below:

**“Figure 1**. Flowchart of patients included in the present study.

**Figure 2**. Nomogram to predict the probability of MES in patients with acute stroke of the middle cerebral artery. According to nomogram points for BZ, SCI < 10 mm, CI. The total points were the sum of the three points, and we can evaluate the risk of MES from the line of Risk of Event according to the total points.

**Figure 3**. Discrimination and calibration assessment of the model. **(A)** ROC curve and AUC of the nomogram in the training cohort. **(B)** Calibration curve for the nomogram to predict the probability of MES+ with bootstrap sampling validation. The bias-corrected curve is plotted by bootstrapping using 200 resamples. The ideal curve is the 45° dashed line, which indicates perfect prediction. **(C)** Calibration curve for the nomogram to predict the probability of MES+ with leave-one-out cross-validation. **(D)** Decision curve for the predictive nomogram. The net benefits were measured at different threshold probabilities. The blue line represents the predictive nomogram. The middle blue line represents the assumption that all patients are MES+. The middle gray line represents the assumption that all patients are MES-.”

The publisher apologizes for this mistake. The original version of this article has been updated.

